# Changes in expression levels of erythrocyte and immune-related genes are associated with high altitude polycythemia

**DOI:** 10.1186/s12920-023-01613-9

**Published:** 2023-07-28

**Authors:** Siwei Feng, Gang Wei, Xuelin Yang, Zhiying Zhang, Jingfeng Qu, Donglan Wang, Tian Zhou, Ting Ni, Lijun Liu, Longli Kang

**Affiliations:** 1 Key Laboratory for Molecular Genetic Mechanisms and Intervention Research on High Altitude Disease of Tibet Autonomous region, Key Laboratory of High Altitude Environment and Genes Related to Diseases of Tibet Autonomous Region, School of Medicine, Xizang Minzu University, Xianyang, Shaanxi 712082 China; 2 Ministry of Education (MOE) Key Laboratory of Contemporary Anthropology, Collaborative Innovation Center of Genetics and Development, Human Phenome Institute, School of Life Sciences, Fudan University, Shanghai, 200438 China; 3 The Second People’s Hospital of Tibet Autonomous Region, Lhasa, Tibet, 850000 China

**Keywords:** Chronic Mountain Sickness, High altitude polycythemia, Transcriptome analysis, Differentially Expressed Gene, Molecular mechanism

## Abstract

**Background:**

As a chronic mountain sickness(CMS) with the highest incidence and the greatest harm, the pathogenesis of high altitude polycythemia (HAPC) is still not fully understood.

**Methods:**

37 HAPC patients and 42 healthy subjects were selected from plateau, and peripheral venous blood samples were collected for transcriptome sequencing on Illumina NovaSeq platform. The sequenced data were analyzed by bioinformatics and phenotypic association analysis.

**Results:**

The results showed significant differences in multiple clinical indicators including RBC and HGB et al. existed between HAPC and control. Based on the RNA-seq data, 550 genes with significant differential expression were identified in HAPC patients. GO and KEGG pathway enrichment analysis showed that the up-regulated genes were mainly enriched in processes such as erythrocyte differentiation and development and homeostasis of number of cells, while the down-regulated genes were mainly enriched in categories such as immunoglobulin production, classical pathway of complement activation and other biological processes. The coupling analysis of differential expression genes(DEGs) and pathological phenotypes revealed that 91 DEGs were in close correlation with in the phenotype of red blood cell volume distribution (width-CV and width-SD), and they were all up-regulated in HAPC and involved in the process of erythrocyte metabolism. Combined with the functional annotation of DEGs and literature survey, we found that the expression of several potential genes might be responsible for pathogenesis of HAPC. Besides, cell type deconvolution analysis result suggested that the changes in the number of some immune cell types was significantly lower in HAPC patients than control, implying the autoimmune level of HAPC patients was affected to a certain extent.

**Conclusion:**

This study provides an important data source for understanding the pathogenesis and screening pathogenic genes of HAPC. We found for the first time that there was a significant correlation between HAPC and the pathological phenotype of width-CV and width-SD, wherein the enriched genes were all up-regulated expressed and involved in the process of erythrocyte metabolism. Although the role of these genes needs to be further studied, the candidate genes can provide a starting point for functionally pinning down the underlying mechanism of HAPC.

**Supplementary Information:**

The online version contains supplementary material available at 10.1186/s12920-023-01613-9.

## Background

The Qinghai-Tibet Plateau, located in southwest China with an average altitude of over 4,000 meters, is the highest and largest plateau in the world. In order to adapt to the long-term hypoxic environment, people need some adaptive changes when entering the plateau area from the plain. However, each individual has his own limit to adapt to the plateau hypoxia. Incomplete adaptation will lead to the occurrence of high-altitude sickness. The indigenous Tibetans on the Qinghai-Tibet Plateau are a unique group, who are the oldest plateau population and have the longest living history, and thus this population have the longest plateau adaptation history and the best adaptation level, which is the result of genetic adaptation obtained by long-term natural selection [1].

Although the indigenous Tibetans on the Qinghai Tibet Plateau may be the most adaptable to high altitude, their protective inactivation of hemoglobin is limited by altitude. For people living at an altitude below 4000 meters, their hemoglobin levels and the prevalence of polycythemia increased linearly with living altitude. Once the living altitude exceeds 4500 meters, due to severe hypoxia, the protective passivation of hemoglobin of the Tibetan people living in the Qinghai Tibet Plateau will become ineffective, and the CMS symptoms will occur, which is a huge live stress for them [2]. As early as 1987, Wu et al. [3] reported in detail 26 cases of indigenous Tibetans suffering from CMS living at an altitude of 3680 ~ 4179m. CMS group had significantly increased number of red blood cells compared with 36 healthy Tibetans living at the same altitude. For people living at an altitude below 4000 meters, their hemoglobin levels and the prevalence of polycythemia increased linearly with living altitude. CMS is a threat to people living on the Qinghai-Tibet Plateau, so it is imperative to prevent and control CMS.

CMS refers to a series of comprehensive high altitude diseases in people living at altitudes above 2,500m for a long time and gradually losing their acclimation to the hypoxic environment [4], including high altitude polycythemia (HAPC), high altitude heart disease (HAHD), high altitude pulmonary hypertension (HAPH), etc. HAPC is of the highest morbidity, the most harmful and characteristic chronic mountain sickness among people living in the plateau [5]. HAPC is characterized by excessive polycythemia and severe hypoxemia. When oxygen receptors in the kidney is stimulated by hypoxia, erythropoietin (EPO) is secreted by renal tubulointerstitial fiber cells to stimulate bone marrow blasts to promote the division of nuclear erythrocytes and accelerate the maturation of red blood cells (RBC), resulting in an increase in the number of RBC in the blood. With the continuous increase in the number of RBC, when the hematocrit exceeds 60%, the blood viscosity increases significantly, which will in turn lead to series of clinical symptoms such as chest distress and chest pain, dizziness, headache, palpitations, shortness of breath, arthralgia and muscle pain, as well as specific signs such as acropachy and plateau red face, and even hinders the transmission of oxygen and aggravates tissue hypoxia. The environment of low pressure and hypoxia at high altitude makes the arterial oxygen partial pressure (PaO_2_) and arterial oxygen saturation (SaO_2_) both decrease, resulting in reduced oxygen delivery to tissues, and may lead to cell hypoxia and organ dysfunction [6]. Chronic hypoxemia triggers a vicious cycle in which abnormal hemorheology burdens the vasculature, which may lead to decreased pulmonary arterial blood flow and dysregulation of the ratio of ventilation and blood flow. Damaged lungs can further increase the degree of arterial hypoxia during gas exchange, which eventually stimulates further RBC production.

The pathological characteristics of HAPC patients can be affected by genetic background, environmental and/or epigenetic factors. The polymorphisms of genetic susceptibility genes play a role in people adapting to high altitude. We had discovered that 12 single-nucleotide variants (SNVS) in 12 genes are associated with the risk of HAPC [7]. Among them, three SNPs of IL12RB1 gene and one SNP of STAT3 gene can be involved in the occurrence of HAPC in Tibetan people living at high altitude[8, 9]. In addition, polymorphisms in genes encoding key hypoxia inducible factors (HIF) such as EPAS1 and EGLN1 were negatively correlated with hemoglobin concentration in population living in the Qinghai-Tibet Plateau[10–12]. The polymorphism in EPAS1 can down-regulate the transcription of EPAS1, and the transcription factor HIF2α encoded by EPAS1 can stimulate the production of RBC, thereby increasing the concentration of hemoglobin in the blood [10]. These results suggest that factors affecting gene expression can be responsible for hypoxia retardation in Tibetan populations.

At present, the pathogenesis of HAPC is not completely clear. In view of this, this study adopted the RNA-Seq method to analyze the gene expression level of HAPC and healthy people through comparative transcriptome studies [13], and conducted correlation analysis of its pathological phenotype and differential genes. In addition, we also tried to link the gene expression difference with the pathological phenotype, attempting to unclose the molecular basis of HAPC pathogenesis from the molecular level and provide reference data for CMS research field.

## Methods

### Study objects and sample collection

A total of 92 volunteers (58 males and 34 females) were recruited in this study, of which, 13 samples were excluded due to the failure of library construction and other reasons, and 79 samples (50 males and 29 females) were finally used for further analysis, including 37 HAPC patients (27 males and 10 females)and 42 healthy subjects(23 males and 19 females). All subjects had lived at altitude above 3600m in Tibet for a long time and had no history of respiratory or cardiovascular diseases. According to the International Consensus Statement (also known as Qinghai Standards) reached by the International Society for Mountain Medicine (ISMM) at the 6th Academic Conference on Alpine Medicine and Plateau Physiology in 2004, the included subjects should meet the criteria of Hb ≥ 21 g/dL in male and Hb ≥ 19 g/dL in female [4].

### RNA sequencing and data processing

The total RNA was extracted from the blood samples of the subjects using PAXgene Blood miRNA Kit(Qiagen, Hilden, Germany). The absorbance values of RNA at 260 nm (A260) and 280 nm (A280) were measured by Nanodrop ND-1000 (Termofisher Scientifc, Waltham, MA), and RNA was evaluated by RNA integrity number (Agilent 2100 RIN Beta software). The RNA-seq libraries were constructed with NEBNext ®Ultra sequencing RNA Library Prep Kit for Illumina ®, and sequenced using Illumina NovaSeq6000 platform to generate paired-end reads. Low-quality reads (the base number of Qphred ≤ 20 accounts for more than 50% of the total read length of reads) were removed, and the high-quality clean data was kept for subsequent analysis. Clean reads were aligned to the human reference genome (GRCh38) using HISAT2 v2.0.5. FeatureCounts (1.5.0-p3) [14] was used to calculate the number of reads mapped to each gene.

### Differentially expressed gene analysis

DESeq2 software (1.20.0) was used to identify the DEGs between HAPC patients (TH) and control population (TC), and Benjamini and Hochberg’s method was used to adjust the resulted P value to control the false discovery rate. The adjusted P values < 0.05 and | log2foldchange | > 1 were used as the criteria to define DEGs.

### Gene enrichment and pathway analysis

Gene Ontology (GO) and KEGG pathway enrichment analysis were performed on differentially expressed genes using clusterProfiler(3.4.4) [15] and Metascape [16]. GO (http://www.geneontology.org) terms were divided into three main classes, i.e., biological processes (BP), cellular components (CC) and molecular functions (MF). KEGG pathway is a collection of manually drawn pathway maps representing the up-dated knowledge of the molecular interaction, reaction and relation networks of multiple biological and pathological aspects, providing a fantastic source for helping understand the roles of DEGs in specific situations. P < 0.05 was considered statistically significant, and the lower the P value, the more significant the enrichment is.

### Correlation analysis between differentially expressed genes and clinical phenotypes

To explore the correlation between RNA-seq data and clinical phenotypes, we first identified differentially expressed genes (DEGs) between the HAPC patients and the controls using DESeq2. Then we extracted the gene expression value evaluated with FPKM (fragments per kilobase of transcript per million fragments mapped). To calculate the correlation extent between the gene expression level and the values of clinical phenotypes, we and normalize both datasets using Z-score normalization method. QT clustering method in MEV package (version 4.9, https://sourceforge.net/projects/mev-tm4/) with the default parameters was used to identify clinical phenotypes that were highly correlated with differentially expressed genes, if a clinical feature and some DEGs are in the same cluster, they are defined as highly correlated or associated.

### Statistical analysis

R software version 4.0.1 (ggplot2 and Immunedeconv software packages) was used to draw heatmap plots, volcano plots and others. The subjects’ clinical characteristic information was compared using independent sample t-test. P-values were adjusted by the false discovery rate, P < 0.05 was considered significant.

## Results

### Differences in clinical characteristics of the subjects

Individuals meeting the Qinghai standards were included in the case group, and normal individuals who lived at the same altitude for a long time were selected as the control group. There were 79 subjects in the two groups, from which 60 types of clinical data were collected. It was exciting to find that multiple clinical phenotypes were significantly different between HAPC patients and the control population, including red blood cell (RBC, P < 0.0001), hemoglobin (HGB, P < 0.0001), hematocrit (HCT, P < 0.0001), mean corpuscular hemoglobin concentration (MCHC, P = 0.0004), platelet (PLT, P < 0.0001), Standard deviation of Red Cell volume Distribution Width (RDW-SD, P < 0.0001), coefficient of variation of Red Cell volume Distribution Width (RDW-CV,P < 0.0001), mean platelet volume (MPV, P = 0.0125), platelet-larger cell ratio (P-LCR, P = 0.0012), plateletcrit (PCT, P < 0.0001), Basophil (BASO,P = 0.0039) (Table 1).

**Table 1:** Clinical characteristics of the study subjects

Characteristic	HAPC patients n = 21	Healthy patients n = 39	*P* value
WBC (*10^9/L)	6. 13(2.71)	5.85(3.65)	0.5929
RBC (*10^12/L)	7.43(0.90)	4.76(0.42)	<0.0001
HGB (g/L)	216.50(23.50)	146.00(19.00)	<0.0001
HCT (%)	66.60(7.73)	44.20(5.00)	<0.0001
MCV (fL)	91.79 ± 6. 13	90.54 ± 3.96	0.0903
MCH (pg)	29.89 ± 2.42	30. 14 ± 1.70	0.5027
MCHC (g/L)	327.00(10.50)	336.00(14.00)	0.0004
PLT (*10^9/L)	151. 13 ± 62.53	217.74 ± 49.98	<0.0001
RDW-SD (fL)	49.55(10.55)	43.70(3.00)	<0.0001
RDW-CV (%)	15.45(2.93)	13.20(1. 10)	<0.0001
PDW (fL)	15.35(2.28)	15.30(3.90)	0.3784
MPV (fL)	11. 15 ± 1.00	10. 15 ± 1.40	0.0125
P-LCR (%)	35.86 ± 7.96	26.27 ± 9.94	0.0012
PCT (%)	0. 15 ± 0.04	0.23 ± 0.05	<0.0001
NEUT (*10^9/L)	3.75(3.45)	6.39(52.45)	0.6199
LYMPH (*10^9/L)	1.56(1.36)	1.90(22.77)	0. 1607
MONO (*10^9/L)	0.57(0.31)	0.44(6. 18)	0.7742
EO(*10^9/L)	0.08(0.20)	0. 15(1.63)	0.0757
BASO(*10^9/L)	0.02(0.03)	0.04(0.28)	0.0039
NEUT(%)	60.22(19. 13)	57.80(60.59)	0.3524
LYM (%)	24.55(18.36)	21.20(28.88)	0.8585
MONO (%)	7.70(4.93)	4.20(6.62)	0.0697
EO (%)	1.10(1.85)	0.80(2. 13)	0.7271
BASO (%)	0.30(0.33)	0.30(0.57)	0.4183

WBC(*10^9/L):white blood cell, RBC(*10^12/L):red blood cell, HGB(g/L):hemoglobin, HCT(%):hematocrit, MCV(fL):mean corpuscular volume, MCH(pg):mean corpuscular hemoglobin, MCHC(g/L):mean corpuscular hemoglobin concentration, PLT(*10^9/L):platelet, RDW-SD(fL): red blood cell volume distribution width-SD, RDW-CV(%): red blood cell volume distribution width-CV, PDW(fL):platelet distribution width, MPV(fL):mean platelet volume, P-LCR(%):platelet -larger cell ratio, PCT(%):plateletcrit, NEUT(*10^9/L):neutrophil, LYMPH(*10^9/L):lymphocyte, MONO(*10^9/L):monocyte, EO(*10^9/L):eosinophil, BASO(*10^9/L):basophil, NEUT(%):neutrophil percentage, LYM(%):lymphocyte percentage, MONO(%):monocyte percentage, EO(%):eosinophil percentage, BASO(%):basophil percentage

The data in the table are denoted as mean ± standard deviation or median (interquartile range). The independent sample t-test is used for significance test on data with normal distribution, and the Mann-Whitney U-test in the nonparametric test is used for data with non-normal distribution. The difference significance between the two groups is denoted by P value.

**Fig. 1:**
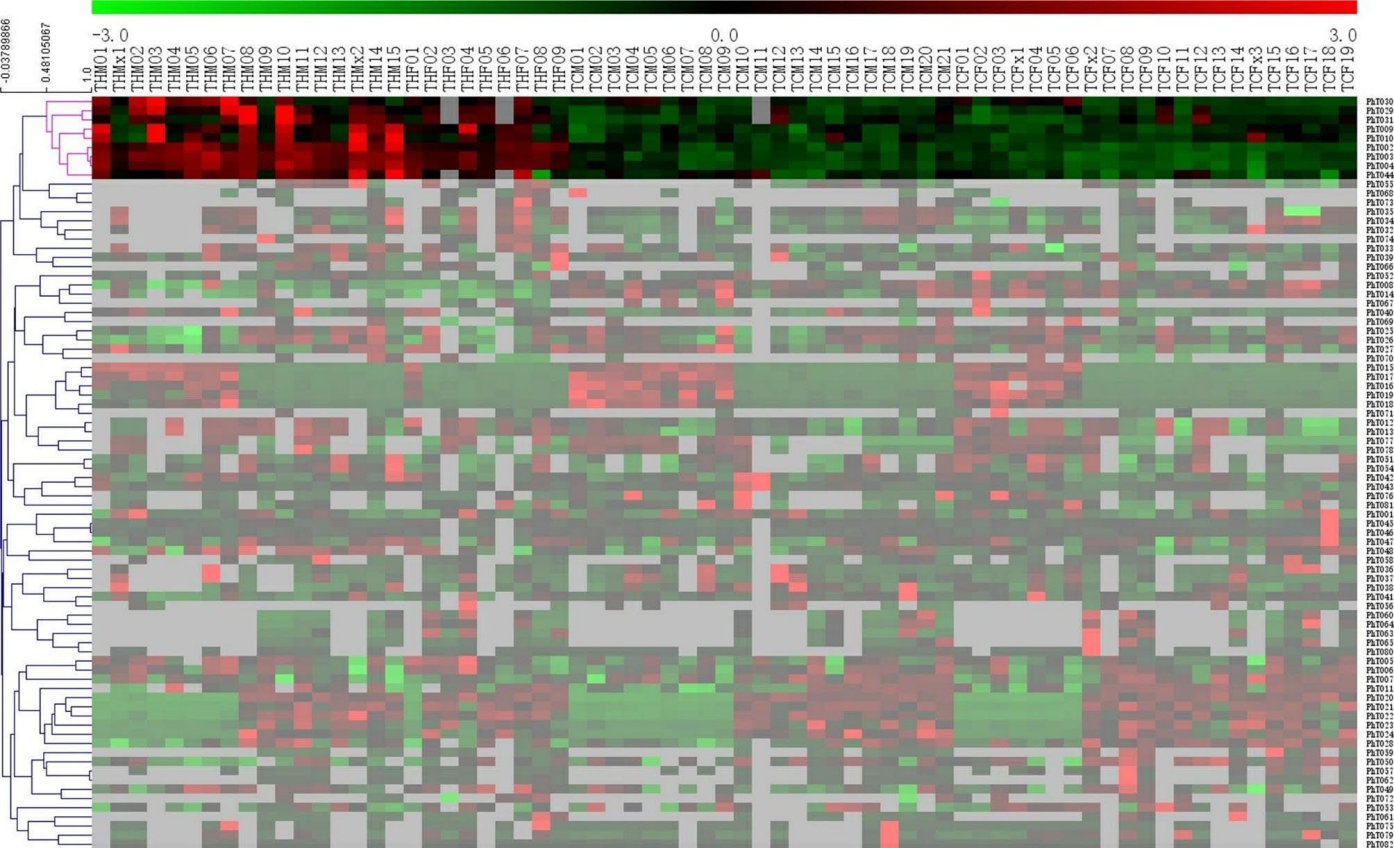
Heatmap of overall similarity of pathological phenotypes. The Abscissa represents the subject, and the ordinate represents different pathological phenotypes

Besides, hierarchical cluster analysis was carried out on Z-score normalized clinical data, The result demonstrated that some clinical phenotypes can clearly separate HAPC patients and the healthy control population, and some of which can be explain well the HAPC pathological phenotypes, such as RBC, RDW-SD, RDW-CV, HGB, MCV, total bilirubin, direct bilirubin, indirect bilirubin, uric acid, adenosine deaminase (Fig. 1). This result was overall consistent with that based on significance test on clinical phenotypes.

### Differentially expressed genes can separate HAPC patients and the control population

**Fig. 2:**
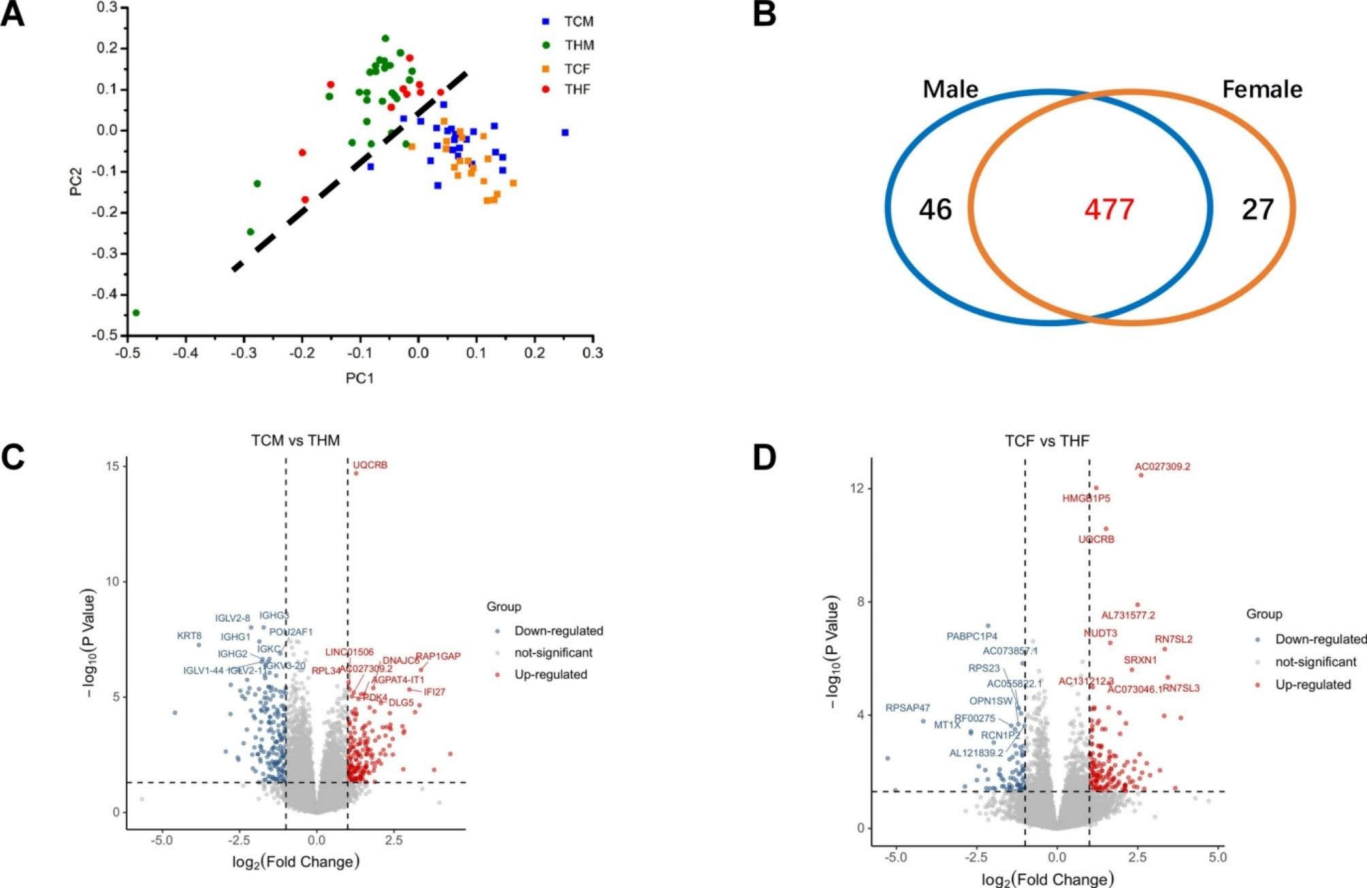
Comparison between HAPC and control individuals. (**A**) PCA map; (**B**) Venn diagram; (**C**) Volcano map of DEGs in males; (**D**) Volcano map of DEGs in females(Grey dotted line indicates the threshold for p < 0.05 and |log2 FC|<1, Blue and red points represent down-regulated and up-regulated DEGs respectively. The top 10 genes are marked in the picture.)

The gene expression profiles of HAPC group and control group were analyzed by DEGs. Figure 2A shows the principal component analysis (PCA) of the sample, which can be clearly divided into TC group and TH group. RNA-seq data identified tens of thousands of expressed genes in 79 PBMC samples.We identified a total of 550 DEGs between HAPC patients and the healthy control population with the criteria of |log2 FoldChange| > 1 and padj < 0.05 (Table S1),among which 477 DEGs (86.7%) were at least different in male or female HAPC samples with the same direction of change between men and women (Fig. 2B and Table S2).

There were 364 DEGs between male HAPC group (THM) and male control group (TCM), including 178 up-regulated genes and 186 down-regulated genes (Fig. 2C). There were 226 DEGs between the female HAPC group (THF) and the female control group (TCF), including 150 up-regulated genes and 76 down-regulated genes (Fig. 2D).Of note, the prevalence rate of HAPC in males is 3.4%, which is higher than that in females (1.9%) [17]. Interestingly, gender differences were also illustrated in our data, that is, the male group had significantly more differentially expressed genes than the female group (364 vs. 226).

**Fig. 3:**
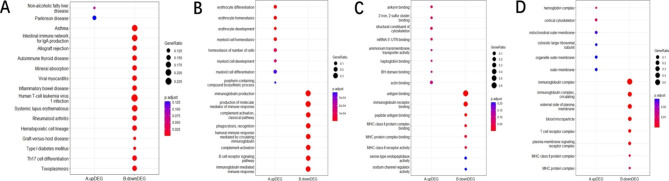
Go and KEGG enrichment analysis of DEGs. (**A**) KEGG pathway enrichment; (**B**) GO: biological process; (**C**) GO: cellular component; (**D**) GO: molecular function (The different colors from blue to red represent the P adjust. The different sizes of the round shapes represent the GeneRatio number in a pathway.)

Due to the great individual differences caused by various influencing factors in test population, in order to obtain a relatively reliable DEG set that can cover both males and females, these 477 DEGs that demonstrated at least in one sex and showed the same differential expression trend were focused for further investigation. The down-regulated genes are mainly enriched in immune-related pathways (Fig. 3A) such as asthma, systemic lupus erythematosus (SLE) and rheumatoid arthritis (RA). In GO analysis, the up-regulated genes are mainly enriched in the biological processes related to erythrocyte cells, such as erythrocyte differentiation, erythrocyte homeostasis, erythrocyte development and homeostasis of number of cells, while the down-regulated genes are mainly enriched in processes related to immune response such as immunoglobulin production, complement activation classical pathway and immunoglobulin mediated immune response and other processes (Fig. 3B). Similar results as described above were also obtained in GO analysis of CC and MF (Fig. 3C,3D).

### DEGs are associated with RBC-associated pathological phenotypes

The change in gene expression is the molecular basis underlying phenotypic differences, thus the characteristic differences between HAPC patients and healthy population can be reflected by the DEGs between them. Interestingly, we found that gene expression differences have good correlations with pathological features. In order to help understand the possible causes underlying pathological phenotypes in HAPC patients, we conducted an association analysis between the normalized values of pathological phenotypes of HAPC and DEGs. The pathological phenotypes and genes enriched in the same cluster indicate a strong correlation between them.

**Fig. 4:**
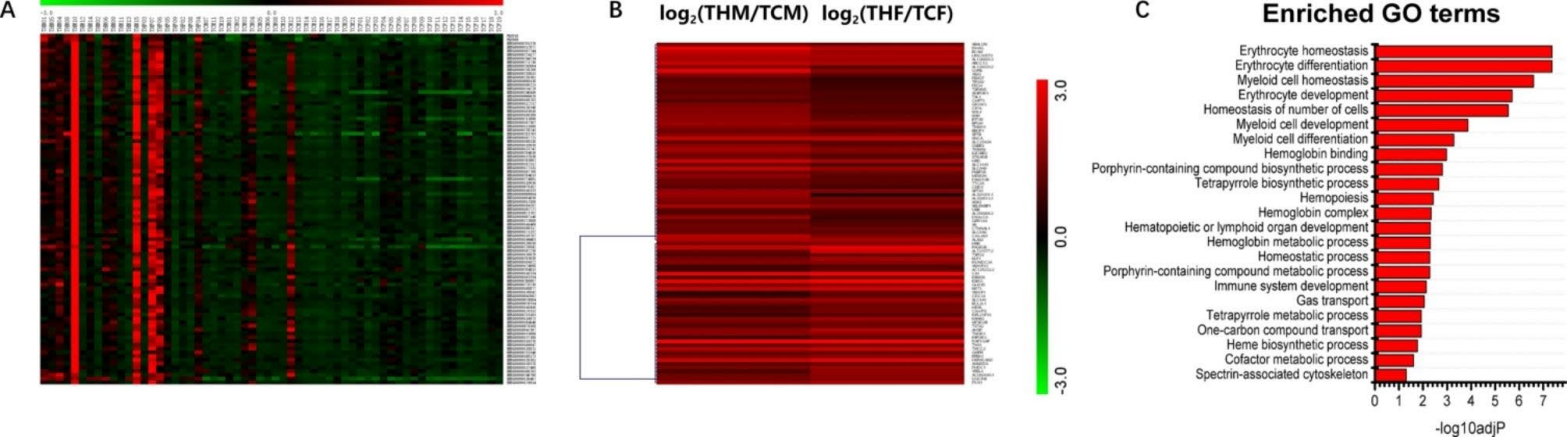
Combined analysis of DEG and pathophenotype. (**A**) Hierarchical clustering Heatmap of phenotype (RDW-CV and RDW-SD) and DEGs (The Abscissa represents the subject, and the ordinate represents pathological phenotypes and gene ID of each DEG in clustering group.); (**B**) Expression of different genders of DEGs in clustering group(The Abscissa represents the comparison between the male and female, and the ordinate represents gene name of each DEG in clustering group.); (**C**) Go analysis of DEGs in clustering group

Through the joint analysis of DEG and pathological phenotype, it was found that under the grouping of erythrocyte-related pathological phenotype-volume distribution width (RDW-CV and RDW-SD), there were 91 DEGs (Fig. 4A), including 75 Protein Coding gene,11 LncRNA, and 5 Pseudogene. Among the genes involved in erythrocyte metabolism, there are many star molecules, such as the candidate gene hemoglobin subunit (hemoglobin subunit beta and delta,HBB and HBD),BPGM (Bisphosphoglycerate Mutase) as a biomarker for hypoxia in vivo, and ALAS2 (5'-Aminolevulinate Synthase) as a target gene for the core transcriptional regulator GATA-1 of erythroid differentiation 2) and other stars. Interestingly, DEGs in this group were all highly expressed in HAPC patients compared with the control (Fig. 4B). GO functional enrichment of genes in this cluster showed that that these genes were enriched in erythrocyte metabolism-related terms such as erythrocyte homeostasis, erythrocyte differentiation, hemoglobin complexes and its metabolic processes (Fig. 4C). So it is clearly that they are centainly responsible for the HAPC-relevant pathological features (RDW-CV and RDW-SD).

In order to screen for the novel genes that play an important role in the pathological phenotypes of HAPC, RDW-CV and RDW-SD, we tried to find genes that function in the process of erythrocyte metabolism or in the environment of low pressure and hypoxia at high altitude. Excluding genes having already been reported in relevant literatures, a total of 6 genes were screened out, including ferrochelatase (FECH), carbonic anhydrase 1 (CA1), glutaredoxin 5 (GLRX5), erythrocyte membrane protein band 4.2 (EPB42), spectrin beta erythrocytic (SPTB), spectrin alpha erythrocytic 1(SPTA1) (Table 2). Of note, CA1 and GLRX5 were also in the list of 40 genes exhibiting significant expression changes and in the same trend in both male and female groups. These six genes are all involved in the metabolism of RBC or the synthesis of hemoglobin, indicating they are good candidate genes responsible for related pathological phenotypes in HAPC patients.

**Table 2:** Part of significantly differentially expressed genes in RBC related pathological phenotypes

gene_ID	Phenotype|geneName_gene Description__TFfamily	log2FoldChange	padj
ENSG00000066926	FECH___ferrochelatase [Source:HGNC Symbol;Acc:HGNC:3647]___-	1.649370052	5.63688E-06
ENSG00000133742	CA1___carbonic anhydrase 1 [Source:HGNC Symbol;Acc:HGNC:1368]___-	3.554716958	1.21851E-10
ENSG00000182512	GLRX5___glutaredoxin 5 [Source:HGNC Symbol;Acc:HGNC:20134]___-	1.639986519	4.5607E-05
ENSG00000166947	EPB42___erythrocyte membrane protein band 4.2 [Source:HGNC Symbol;Acc:HGNC:3381]___-	1.717224871	0.001147742
ENSG00000070182	SPTB___"spectrin beta, erythrocytic [Source:HGNC Symbol;Acc:HGNC:11274]"___-	1.814173639	1.09875E-05
ENSG00000163554	SPTA1___"spectrin alpha, erythrocytic 1 [Source:HGNC Symbol;Acc:HGNC:11272]"___-	2.137330642	2.13008E-05

### Decreased immune level is a novel feature in HAPC patients

**Fig. 5:**
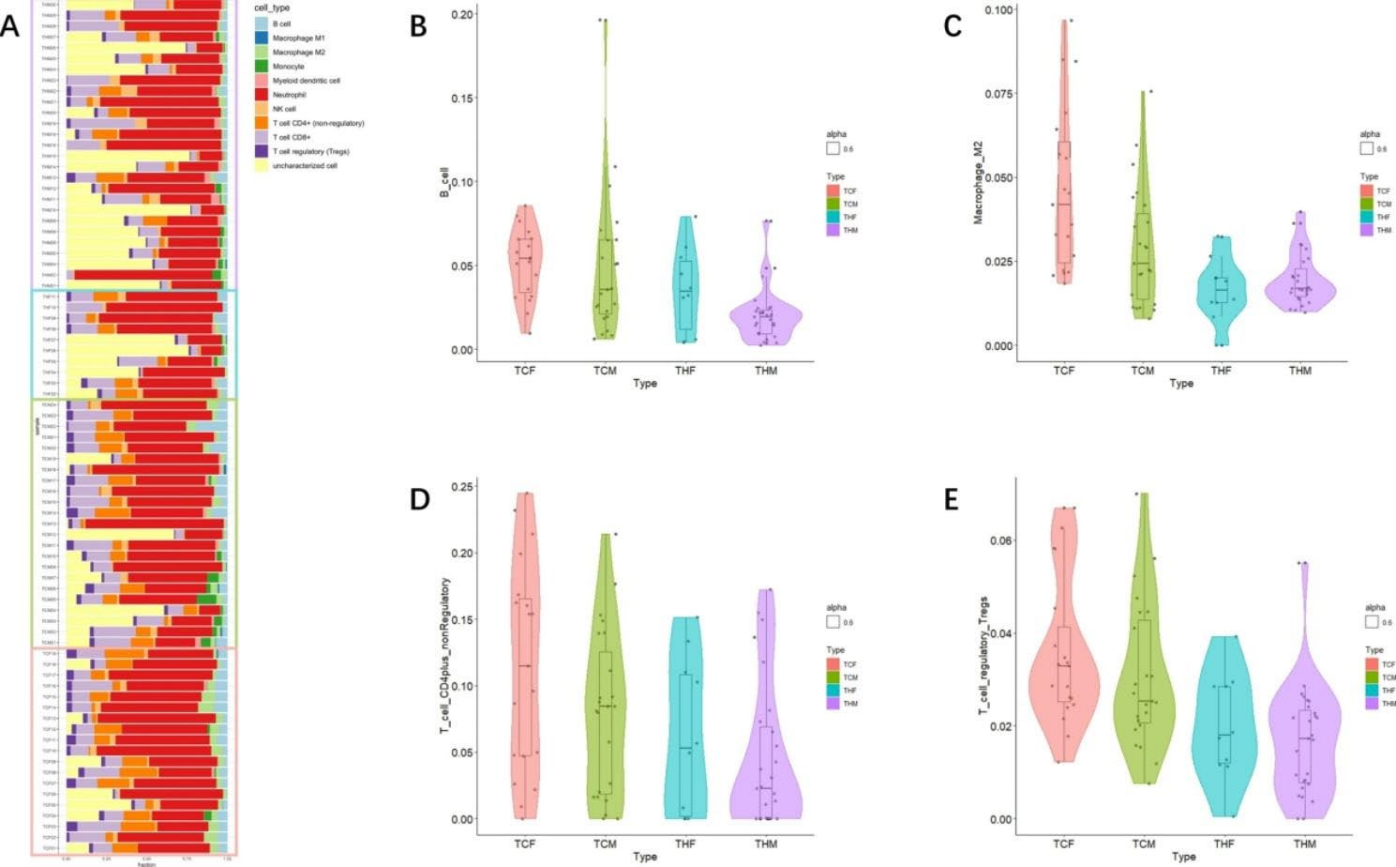
Analysis of pathological phenotype related to immune response. (**A**) Proportion of various immune cells in each subject (Light blue indicates B cells, blue indicates Macrophage M1,light green indicates Macrophage M2, green indicates Monocyte, pink indicates Myeloid dendritic cell,red indicates Neutrophil,light orange indicates NK cell,orange indicates T cell CD4+(non-regulatory),lilac indicates T cell CD8^+^,purple indicates T cell regulatory (Tregs),yellow indicates uncharacterized cell.);(**B**) Expression of B cells in each group; (**C**) Expression of M2 macrophages in each group; (**D**) Expression of CD4^+^_nonRegulatory T cells in each group; (**E**) Expression of Tregs cells in each group

As described above, down-regulated DEGs were mainly enriched in immune-related functional categories (Fig. 3A,3B), indicating some kind of immune abnormalities or even disorders existed in HAPC patients. In order to further reveal the relationship between immunity and HAPC, we estimated the composition of different types of immune cells in blood samples based on the RNA-Seq expression profile data of each sample with Immunedeconv (v0.1.0) [18]. The results show that the proportion of immune cell types varied in test samples (Fig. 5A). There were significant decreases in the proportions of four of immune cell types in HAPC patients compared with the control population, which are B cells (Fig. 5B), Macrophage_M2 (Fig. 5C), T_cell_CD4plus_nonRegulatory (Fig. 5D) and T cell_regulatory_Tregs (Fig. 5E). This result is consistent with the result that down-regulated genes in HAPC patients were mainly enriched in the immune-related pathways, which also implied that the reduction of these four immune cell types in the blood of HAPC patients may be relevant to the pathological features or the pathogenesis of HAPC patients, which is worthy of further investigation.

## Discussion

HAPC is a chronic high-altitude disease when adapting to high altitude, which exhibits with increases in red blood cells (RBC) and the concentration of hemoglobin (HGB) [19], and seriously threatens the health of people on plateau. Therefore, this study explored the differences in gene expression between HAPC patients and control populations by transcriptome profiling on the peripheral blood samples using RNA sequencing method, attempting to make a step forward in understanding the molecular basis of HAPC and discovering potential novel candidate genes with the potential to characterizing or treating HAPC.

Through series of bioinformatics analysis and literature searching, we finally screened out 6 novel candidate genes (FECH, CA1, GLRX5, EPB42, SPTB, SPTA1) that may be involved in the development of physiological or pathological conditions in HAPC patients. Based on the annotated function and reported biological roles of these genes, they have the potentials to play important roles in HAPC pathological conditions. For example, FECH is the last enzyme in heme biosynthesis pathway, and plays an critical role in heme biosynthesis [20]. Heme as the auxiliary group of hemoglobin, causes affecting its synthesis would in turn influence the synthesis of hemoglobin, and finally contribute to the physiological conditions of HAPC patients. CA1, the gene encoding Carbonic Anhydrase 1, is an intracellular enzyme in human red blood cells (RBC), the inhibition of its activity, by chemical reagents such as acetazolamide [21] and mezolamide [22], can increase the systemic metabolic acidity and improve ventilation and oxygenation level, these chemicals are now widely used as drugs for the prevention and treatment of acute mountain sickness. In addition, the increased expression of CA1 can lead to decreased oxygenation level, which may lead to severe erythrocytosis and HAPC symptom. GLRX5, the gene encoding glutaredoxin 5, a mitochondrial protein involved in [Fe-S] cluster biosynthesis and required for normal iron homeostasis in human cells, its decreased expression can cause the significantly reduced iron chelatase level and the increased transferrin level [23]. We found that the increased GLRX5 expression in HAPC patients is likely to lead to an increase in [Fe-S] cluster synthesis, which in turn affect the hemoglobin biosynthesis in associated cells. Of note, the deficiency or mutation of the genes such as EPB42, SPTB and SPTA1 can cause hereditary polycythemia [24], 25], which is also an important cause of hemolytic anemia. In particular, the change of SPTA1 mRNA expression also affects the survival time of RBC [26]. So, combined these facts described above, it is speculated that the increased expression of FECH, CA1 and GLRX5 may be some of the characteristic changes in HAPC patients. The detailed functions and the potential contributions of these candidate genes will be systematically investigated in the future.

The 40 DEGs differentially expressed in the same direction in both male and female HAPC patients were enriched in the pathway of Cell Response to Copper Ions. It has been reported that intracellular copper ions can interact with hemoglobin to form methemoglobin by direct electron transfer from heme Fe^2+^ to Cu^2+^ [27]. Similar results were found in another study, wherein metal ions of zinc, copper and iron can affect the activity of erythrocyte carbonic anhydrase (CA) and hemoglobin content. Copper ion strongly inhibits erythrocyte CA [28], compared with hemoglobin, CA is more sensitive to zinc and copper ion. Therefore, we speculate that these differentially expressed genes in the Cell Response to Copper Ions pathway potentially play some roles in the occurrence and development of HAPC symptoms, which is worthy of further investigation.

The present study also showed that the proportion of four immune cell types were significantly decreased in HAPC blood samples compared to the control ones(Fig. 5), suggesting that changes in immune system may be another feature of HAPC and might also contribute to the sickness in HAPC patients. High altitude acclimatization can affect the immune system. Studies have shown that long-term or short-term exposure to Qinghai-Tibet Plateau can inhibit the adaptive immune response mediated by lymphocytes, but this response may recover to some degree with the increase of exposure time [29]. The adaptive immune system depends on B and T cells. Interestingly, we also found that down-regulated DEGs were enriched in two immune-related pathways: "Adaptive immune response" and "Positive regulation of B cell activation". In vitro experiments have shown that the adaptive immune response in humans depends on oxygen levels [30]. HAPC patients have different expression patterns from other populations. A study conducted at an altitude of 3700m showed that neutrophils increased, lymphocytes decreased slightly [31], and neutrophils increased slightly after exposure to acute hypoxia [32]. Hypoxia preconditioning combined with altitude training increases the expression of CD4/CD8 on T lymphocytes [33]. Recent cytological evidence suggests that metabolic stress caused by hypoxic conditions rapidly leads to T-cell depletion [34]. These above surveys showed contradictory results, however, in the comparison between HAPC and healthy control group in our study, there was no significant difference in white blood cells containing multiple immune cell types between the two groups (Table 1, P = 0.59). Such results suggest that the decline of immune level is probably caused by the environment of low pressure and hypoxia at high altitude, rather than the cause of HAPC.

This study has certain limitations. When evaluating the results, the limited sample capacity may cause errors to the results. In the future, we will use in vitro models or Ingenuity Pathway Analysis [35] to evaluate the genes or pathways identified here and further study the mechanisms of HAPC development.

## Conclusion

In summary, by comparative transcriptome profiling on the peripheral blood of HAPC patients and healthy control people, we found for the first time that HAPC patients were featured by significant changes in series of clinical phenotypes including red blood cell volume distribution width, correspondingly, the differentially expressed genes were enriched in the biological process of erythrocyte metabolism and were all up-regulated in HAPC patients. Although the role of these genes in HAPC needs further study fully understand the pathogenesis mechanism of HAPC, these candidate genes provide a starting point for further exploration of functional genes in HAPC. This study also provides an important data source for understanding the pathogenesis of HAPC and screening for relevant pathogenic genes in population living at high altitude.

## Electronic Supplementary Material

Below is the link to the electronic supplementary material

Supplementary Material 1

Supplementary Material 2

Supplementary Material 3

Supplementary Material 4

## Data Availability

The data presented in the study are deposited in the Genome Sequence Archive in National Genomics Data Center, China National Center for Bioinformation/Beijing Institute of Genomics, Chinese Academy of Sciences (GSA-Human:HRA004467) that are accessible at https://ngdc.cncb.ac.cn/gsa-human.

## Abbreviations

DEG: Differential expression gene
HAPC: High Altitude Polycythemia
CMS: Chronic mountain sickness
RBC: Red blood cells
HGB: Hemoglobin
PCA: Principal component analysis
GO: Gene Ontology
BP: Biological process
CC: Cellular component
MF: Molecular function
KEGG: Kyoto Encyclopedia of Genes and Genomes
